# Complete mitochondrial genome of an Asian longicorn beetle, *Olenecamptus bilobus* Fabricius (Coleoptera: Cerambycidae: Lamiinae)

**DOI:** 10.1080/23802359.2021.1875897

**Published:** 2021-02-12

**Authors:** Zishu Dong, Xiaoyun Wang, Yueshi Li, Daming Huang, Wen Lu

**Affiliations:** a Guangxi Key Laboratory of Agric-Environment and Agric-Products Safety, National Demonstration Center for Experimental Plant Science Education, College of Agriculture, Guangxi University, Nanning, China; b Guangxi Wuzhou Rural Investment Development Co., Ltd, Wuzhou, China; c Qingxiushan Scenic Spot Tourism Development Co, Ltd, Nanning, China

**Keywords:** *Olenecamptus bilobus*, mitochondrial genome, phylogenetic tree, Subject classification codes: *Olenecamptus bilobus* Fabricius

## Abstract

*Olenecamptus bilobus* Fabricius is widely distributed in some parts of Southeast and East Asia whose larvae bore under the bark of at least eleven plant families. The complete mitochondria genome of *O. bilobus* was 15,262 bp in length, with 37 genes, including 12 protein-coding genes (PCGs), 23 tRNA genes (tRNAs), and 2 rRNA genes (rRNAs). The A + T content is 76.91%, showing strong AT skew. Phylogenetic analysis indicated that *O. bilobus* had a close relationship with *Olenecamptus subobliteratus* Pic.

## Introduction

*Olenecamptus bilobus* is a common pest whose larvae bore under the bark of at least eleven plant families such as mulberry, willow, poplar, oyster, oak, sassafras, tussah, maple, banyan, claw beetle, pineapple etc. (Chen et al. 1959). According to the few reports in the past, we can know that *O. bilobus* widely distributed in some parts of Southeast and East Asia (Hua et al. 2009; Saha et al. 2013). *Olenacamptus taiwanus* Dillon & Dillon was once mistaken as a subspecies of this species which reflected the necessity of mitochondrial genome for species identification (Wang and Tang 2018 Wang et al. 2019; Su and Wang 2020; Zhou et al. 2020). Therefore, it is necessary to study the complete mitochondrial DNA (mtDNA) genome of *O. bilobus* which provides a theoretical basis for future research on *Olenecamptus* chevrolat.

In this study, specimens of *O. bilobus* were collected from the Qingxiu Mountain (22°47’N, 108°23’E) of Nanning City (Guangxi Autonomous Region, China) on a banyan tree. The total genomic DNA was extracted following the modified CTAB DNA extraction protocol and stored at Guangxi Key Laboratory of Agric-Environment and Agric-Products Safety (The city of Nanning, China) with sample number of SZHT0606G148. Then library was constructed and pair-end was sequenced (2*150 bp) with HiSeq (Illumina, San Diego, CA). Approximately 11.10 G of raw data and 11.00 G of clean data were obtained for sequence assembly by SPAdes (version 3.9) (Bankevich et al. 2012). MITOS (http://mitos.bioinf.uni-leipzig.de/index.py) was used for mitochondrial genome annotation (Bernt et al. 2013).

The complete mitochondrial genome of *O. bilobus* is a closed circular molecule 15,262 bp in length (GenBank accession number MT740324) and constitutive of 37 genes. These genes contain 12 protein-coding genes (PCGs), 23 transfer RNA (tRNA) genes, 2 ribosomal RNA (rRNA) genes, and 1 A + T region (D-loop). The single A + T region is 613 bp in length. The nucleotide composition of the *O. bilobus* mitogenome was A (38.36%), T (38.55%), G (9.24%), C (13.85%). The A + T content is 76.91%, showing strong AT skew.

**Figure 1.**
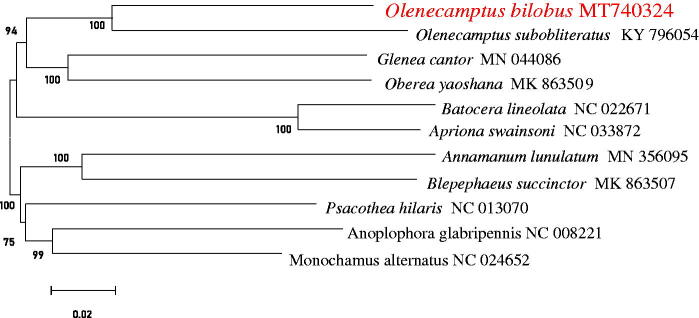
Neighbor-Joining phylogenetic tree of *O. bilobus* and some other Lamiinae beetles. The complete mitochondrial genome was downloaded from GenBank and the phylogenic tree was constructed by Neighbor-Joining method with 1000 bootstrap replicates were estimated using the MEGA 7.0.

Molecular Evolutionary Genetics Analysis Version 7.0 (MEGA 7.0) was used to make phylogenetic analysis among Lamiinae species by Neighbor-Joining method with 1000 bootstrap replicates (Kumar et al. 2016). The results showed that mtDNA of *O. bilobus* had a close relationship with that of *O. subobliteratus* (Figure 1).

## Data Availability

Mitogenome data supporting this study are openly available in GenBank at: https://www.ncbi.nlm.nih.gov/nuccore/MT740324. Associated BioProject, SRA, and BioSample accession numbers are https://www.ncbi.nlm.nih.gov/bioproject/PRJNA663376, https://www.ncbi.nlm.nih.gov/sra/SRX9145194, and SAMN16131500, respectively.
